# Criminal Abortion

**Published:** 1874-07

**Authors:** James S. Whitmire

**Affiliations:** Metamora, Ill.


A MONTHLY RECORD OF

*Medicine, Surgery and the Collateral Sciences.*

EDITED BY J. ADAMS ALLEN, M.D., LL.D.; AND WALTER HAY, M.D.

VOL. XXXI. — JULY, 1874.—No. 7.

### Original Communications.

ARTICLE I. — *Criminal Abortion.* A paper read before the Woodford County Medical Society at its Special Meeting in Eureka, on Tuesday, October 21st, 1873. By JAMES S. WHITMIRE, M.D., of Metamora, Ill.

*Mr. President and Members of the Association:*

Having been appointed at a previous session of this Society, to make a special report upon the subject of Criminal Abortion, I respectfully beg leave to submit the following.

Before entering upon my subject, I would remark that there seems to be a growing indisposition on the part of American females, married or unmarried, to bear children, and that when children are born, legitimate or illegitimate, they are, by a large proportion of our female population, considered more as a burden and an impediment to the pleasure and enjoyment of the houi, than as a great and lasting blessing, given to us in a bundle of rollicking humanity for our safe keeping and care, as a source of infinite pleasure and happiness.

Abortion may occur from a great variety of causes, among which may be reckoned, nervous excitability, plethora, blows or falls, great exertion and fatigue, sudden fright and other violent emotions of the mind; too sparing a diet, or a diet that is too nutritious, producing a full habit, or plethora; the abuse of alcoholic drinks, fevers of a low grade, exhausting hemorrhages, colic from accumulated fæces, immoderate venery, etc.

This accident is nearly or quite always preceded by flooding, pain in the back and loins, and it may occur without any obvious cause from some defect in the uterus, or in the fœtus itself, which we are not able to satisfactorily explain; hence, it will not unfrequently occur in the same female at a particular period of pregnancy, and may, perhaps, be kept up, from the influence of habit alone, when all the functions of the reproductive organs are in a perfectly normal condition.

These cases, however, are those of accidental abortion, which are of frequent occurrence in the practice of every medical man, and with which in this paper we have nothing to do, save a simple mention of the ordinary causes of this serious occurrence; serious, because there is nothing that befalls a female, in the course of her life, that is so likely to ruin her health, and consequently destroy her previously sweet and equable temper, depress her spirits, and render her morose and fretful; hence, it is too frequently the entering wedge to domestic infelicity.

But we have now to deal with criminal abortion, both in regard to its effects upon the moral and physical well-being of individuals and its disastrous consequences and demoralization of communities that are afflicted with this *non-infanto* mania. This special criminality is not confined to any particular class or condition in life: it belongs to, and is practiced by, the married and the unmarried; the rich and the poor; the learned and the unlearned: besides, even those who possess a respectable standing in the church, and are apparently, from all outward appearances, patrons of morality, are not exempt from the evil practice of producing abortion for the sole purpose of getting rid of responsibilities that may come to disturb their secular pleasures or to increase the family expenses. It is true that I am making broad assertions, and bringing grave charges against a large and hitherto respectable class of persons, that would startle the well-meaning, but innocent and uninitiated, and cause them to lose faith in their kind, but it cannot be avoided; it is nevertheless true, whitewash it as you will.

As previously mentioned, our American women, especially, think more of the pleasures of the hour, and seem more willing and determined to devote themselves to them than they do to accomplish the holy destiny for which they were created, and to fill, with both pride and pleasure, the physiological condition of their being; hence, comes this criminality, this lax condition of public and private morals in regard to the bearing and rearing of children.

In this fast day and age we do not take time to think of the rebuke, which has become historical, that an old Roman matron gave to a foreign princess then on a visit to Rome. The young princess, among many other inquiries, asked the old lady about her jewels, and wished very much to see them. "With great pleasure I will show them to you," said the matron. Just at this moment the old lady's twelve sons entered the apartment, when the brave old lady, with supreme pride and pleasure depicted in every feature, turned to the princess and said, "These twelve noble boys are my jewels; they will be of service to the State." So, too, it was with our old Revolutionary mothers; when they had sent their husbands and sons to join Washington's army, they gave thanks to God every day that they had been permitted to bear and rear so many noble boys who were willing to defend their individual as well as the colonial rights. But things have greatly changed since then, and the physiological condition of gestation has come to be looked upon as a great burden and grievous to be borne; hence, the prevalence of criminal abortions.

Besides those females who produce abortion upon themselves, there are professional abortionists of both sexes in all our considerable cities, who, for a consideration, undertake to produce abortion in any case presented to them without asking any questions, and it is only when some of their unfortunate victims succumb under the operation, that inquiries are made, the guilty parties found out, and caught and punished according to their just deserts. But, notwithstanding this danger, this nefarious business is carried on to an alarming extent, and both women and men, knowing these things and the probable certainty of relief with but little immediate danger, are more disposed to be loose in their habits and morals than they otherwise would be; because, with the unmarried, in cases of *accident*, they are enabled to get rid of their incumbrances, with but little or no risk of their shame being published, but, nevertheless, their own self-respect is greatly reduced, and their former confidence in and regard for the innocence and virtue of those of their own sex is completely destroyed; and so it continues; from a high-toned vice in the beginning, it subsides into profligacy, and from the latter, it very speedily descends into debauchery, where their days are numbered, and will soon be ended in the most destitute and wretched misery.

In regard to professional abortionists, some of them, both male and female, claim a membership in the profession of medicine, and do a very considerable, lucrative practice, but avarice is their besetting sin, and they have permitted the love of money to carry away their reason, conscience and common sense, so that, regardless of their once good morals, and instincts of their better nature, they have abandoned themselves to vice and become the worst enemies of society. But professional men and women, who are, in fact, educated and qualified in their calling, seldom or never engage in this crime against nature; they have too much professional pride to be bought with gold, or to be influenced by sympathy to be engaged in any such disreputable practices; besides, the moral standing of a professional man should be above reproach or even suspicion.

There are instances in which it may become a matter of serious consideration whether it is not best to produce abortion, or to even bring on premature labor, for the preservation of the mother's life, but this would be legitimate practice, and hence does not come within the scope of this paper. The professional abortionist, however, has nothing of this kind to consider. It is his business to get rid of the embryo or fœtus as the case may be, at all hazards, but the mother's life must be saved for fear of detection in his nefarious business; he and his business are generally known by the lewd of both sexes for miles around where they reside, and also, known to one another in the different cities, so that if a case is likely to be too close at home for complete safety to all parties, the victim is sent to the next bloody station where the operation is performed, and a reciprocity of iniquity is kept up. If a case should at any time prove fatal, as a safety measure, lest the crime be tracked to its hole, the body is mangled and dismembered so that it cannot be recognized, boxed up and sent by rail or on shipboard to some distant point where all trace of the perpetrators of the crime is lost. Occasionally, however, through our detective system, the animal is hunted down, and that justice meted out to him which should follow the perpetration of any crime. Persons who engage in this crime, whether they are professional or self-abortionists, have lost all the natural instincts of humanity; they have neither principle nor good morals, and are, hence, an eyesore to society, a plague-spot upon communities where they exist—lepers, whose infectious breath undermines the very foundation of the morals of the people, and should not be tolerated for a single day, when and where they are known.

The manufacturer, advertiser and even the vender of *Professor Bombasticus' Female Pills*, should be held to a strict accountability for any injury produced by the nostrums they manufacture or vend, because the very tone of their advertisements is intended to call attention to the abortive quality of their drugs. For instance the advertisements run in this way:

FOR SALE.—Professor Bombasticus' FEMALE Pills. They correct all irregularities of females, purge the blood of all impurities, and restore the secretions, whatever the causes of their obstruction.

N. B. Ladies in an interesting situation should avoid them.

Now, the latter N. B. portion of the advertisement is the very point that they wish noticed; it is that which gives to these pills their only notoriety and makes them salable as an abortive remedy, and gives to the self-abortionist her only inducement to purchase them.

The immediate effect of these pills is a violent drastic catharsis; they may, during their disturbance of the system, from their drastic qualities, bring on abortion or premature labor which may be accomplished just as certainly by any of the drastic cathartics, or any other cause that produces a functional disturbance of the general organism. These pills not only depress the vital forces of the general system by their violent action, but they are apt to leave all the functions of the organism in a pathological condition. There is no less criminality, neither is there less responsibility in the act of producing abortion by one's own hand, or by means that are self-devised, in order to escape responsibilities of one kind or another, than there is to those who make the business of producing abortion a matter of merchandise for the accomplishment of sordid ends; and, in either case, the moral sensibilities, and obligations of the individual to society seem to be so blunted as to make them really dangerous members of society, not, probably, from any overt act, or misdemeanor that will be perpetrated by them, but from the corrupt influences that are apt to follow in the wake of such criminals and their crimes; therefore, when either of these classes are overtaken in their iniquity, an example should be at once made of them for the benefit of those who come after them.

When it is known that it is always necessary to use means, more or less violent in their action, to produce abortion, it would be an easy matter to perceive the disastrous constitutional consequences produced upon the habitual self-abortionist. You see her *tottering*, not walking—pale, discouraged, sallow and languid—along your streets; she has prolapsus uteri, probably displacement of the os uteri; her sexual organs are in a flaccid condition; she has profuse leucorrhœa; is afflicted with menorrhagia at her menstrual periods; in short, her health is virtually gone, broken down by those violent means used either by herself or others for the purpose of producing abortion.

And now, in regard to those *female* pills, so extensively advertised, the man who makes and he who vends them should be held criminally liable, by the law, for any damages from abortion or otherwise, that they may produce. Though there is now a law upon our statute books making it penal, or at least a misdemeanor for any druggist, or apothecary, to retail medicines that have the reputation of producing abortion, yet you may step into any country or city drug store in the State of Illinois, and procure, without the prescription of a physician, any one or all of these reputed abortion-producing medicines, not excepting ergot, bark of the root of the cotton-plant, oil of savin, or even the celebrated and never-failing Bombasticus' *female* pills, none of which are called for by individuals who do not belong to the medical profession, without there is intended mischief ahead.

When abortion occurs, even, without the use of these violent drugs, it is very wearing on the naturally delicate female organism, but if it is produced by violence, even though the operation be performed by the most skillful hand and by the most approved method, the consequences may be none the less hazardous. And when it is produced by an ignoramus or bungler, it is positively dangerous, the woman's life is immediately jeopardized, and her health and usefulness are more than likely to be ruined. All these consequences may not immediately follow, nor be at once apparent, but *time*, that develops all things, will certainly tell the sad story of premature decay, and finally, death, the destroyer of all organisms, will make its certain approach.

The reason for these inevitable results is patent enough to those who are acquainted with the laws that govern the life and health of the animal economy. Gestation being a physiological condition, what woman could possibly be in a healthier state than when she is performing one of the greatest and noblest physiological functions of her being? Abortion, from whatever cause produced, is a pathological condition, and its results are pathological, whether they be present or remote, and hence, nothing but constitutional infirmities and premature decay must and will as inevitably follow this condition as any other effect will certainly follow its philosophical cause.

At this moment I can recall to memory many females, who, I have reason to believe, short of absolute knowledge, have, frequently and again, produced abortion upon themselves, sometimes by violent purgation, lifting, jumping, and even with a stiletto, blunt probe, or uterine sound, either of which they have learned to use by reading obscene books published expressly to impart such information. In such cases, if there was but little hemorrhage they would take the chances, and get through in the best manner they could, but if danger occurred on that account, or extraordinary delay, the family physician was called in to complete the job, and the woman in her great simplicity and extreme innocence would tell him of an accidental fall, a hard day's work, or some extraordinary excitement, or other cause for the accident, but the case now being past remedy, his only and sole duty is to remove the embryo or fœtus as soon as practicable and stop the wasting of the crimson current of the precious life.

This is not an isolated case of this kind; such cases exist, everywhere; they are of frequent occurrence in every village and neighborhood. This iniquity is not confined wholly to our populous cities, but is practiced all over our country to an alarming extent, and even the rural districts are not exempt from this criminal practice. Many, indeed, argue, that the practice is not, in fact, criminal, because, they argue, that the child is not viable until the seventh month of gestation, hence there is no destruction of life. The truly professional man's morals, however, are not of that easy caste, because he sees in the germ the probable embryo, in the embryo the rudimentary fœtus, and in that, the seven months viable child and the prospective living, moving, breathing man or woman, as the case may be.

This iniquitous profligacy of human life and prevalent violation of the laws of our being is one great cause and reason for so few native-born children of American parents, according to the number of young married people, and in comparison with those of other nationalities among us. This, too, accounts for so few household gods, to be worshiped by American parents, that would make home happy and overflowing with joy and gladness. This, too, is one of the many reasons why we are fast losing our national characteristics, and slowly merging into those of our foreign population, who, according to the United States statistics of 1870, are rearing fifty per cent. more children according to their number than Americans are doing.

This being the case, I would ask, is it not high time to strike at the evil, wherever and under whatever circumstances it may be found? It seems to me, as an individual, and laying aside the moral bearings of this subject, that the loss of our national characteristics by the gradual merging into those of other peoples of foreign nationalities, of itself would be and will eventually prove a great calamity, and we may well fear and tremble for the future of our country.

The criminality of this thing attaches in two particulars: First, the destruction of human life, and secondly, by destroying the health and vigor of our women, and incapacitating their powers of reproduction, shortening their lives and rendering them incapable of bearing strong, vigorous and healthy children; and certain degeneracy of our race would long since have occurred, had it not been for the continued admixture of our native Puritan and Huguenot blood with that of the strong, robust and healthy foreign element, the individuals of which are making happy and frugal homes among us. Criminality, therefore, attaches not alone to the professional abortionist, but to the self-abortionist as well; and no less criminal is the druggist who sells and the manufacturer who puts up nostrums to be thrown upon the market, with abortion as their salient point to direct attention.

These are all facts, and the conclusions inevitable and incontrovertible; this is known to every professional man, but what are we to do? Is the evil beyond our reach? Certainly the whole of the evil we cannot avert, but we may add our mite and put forth our hand to stay this monster iniquity. This is not the work of the medical profession alone, but the church, the bar, our law-makers, and all good men and women, should engage in it for the benefit of public morals, for the preservation of our national characteristics, and the general well-being of our people. It is a labor of love that we owe to ourselves, to society, and more especially to the opposite sex; and no less to the health and general well-being of mankind.

